# PERM Hypothesis: The Fundamental Machinery Able to Elucidate the Role of Xenobiotics and Hormesis in Cell Survival and Homeostasis

**DOI:** 10.3390/ijms18010165

**Published:** 2017-01-15

**Authors:** Salvatore Chirumbolo, Geir Bjørklund

**Affiliations:** 1 Department of Neurological and Movement Sciences, University of Verona, Verona 37134, Italy; 2 Council for Nutritional and Environmental Medicine, Mo i Rana 8610, Norway; bjorklund@conem.org

**Keywords:** mitochondria, proterome, reactive oxygen species (ROS), oxidative stress, flavonoids

## Abstract

In this article the Proteasome, Endoplasmic Reticulum and Mitochondria (PERM) hypothesis is discussed. The complex machinery made by three homeostatic mechanisms involving the proteasome (P), endoplasmic reticulum (ER) and mitochondria (M) is addressed in order to elucidate the beneficial role of many xenobiotics, either trace metals or phytochemicals, which are spread in the human environment and in dietary habits, exerting their actions on the mechanisms underlying cell survival (apoptosis, cell cycle regulation, DNA repair and turnover, autophagy) and stress response. The “PERM hypothesis” suggests that xenobiotics can modulate this central signaling and the regulatory engine made fundamentally by the ER, mitochondria and proteasome, together with other ancillary components such as peroxisomes, by acting on the energetic balance, redox system and macromolecule turnover. In this context, reactive species and stressors are fundamentally signalling molecules that could act as negative-modulating signals if PERM-mediated control is offline, impaired or dysregulated, as occurs in metabolic syndrome, degenerative disorders, chronic inflammation and cancer. Calcium is an important oscillatory input of this regulation and, in this hypothesis, it might play a role in maintaining the correct rhythm of this PERM modulation, probably chaotic in its nature, and guiding cells to a more drastic decision, such as apoptosis. The commonest effort sustained by cells is to maintain their survival balance and the proterome has the fundamental task of supporting this mechanism. Mild stress is probably the main stimulus in this sense. Hormesis is therefore re-interpreted in the light of this hypothetical model and that experimental evidence arising from flavonoid and hormesis reasearch.

## 1. Introduction

PERM (Proteasome, Endoplasmic Reticulum and Mitochondria) is a new concept introduced to define a functional structure, the “proterome”, described in this review, which should regulate the survival decision of the cell upon oxidative stress. This structure is fundamentally a functional setting of three major components existing within the living cell, namely the mitochondria, the endoplasmic reticulum (ER) and the proteasome, associated also with the activity of peroxisomes, which altogether participate in the complex stress response of cells. The main activity of the proterome is to coordinate and modulate two fundamental mechanisms in cell survival, i.e., apoptosis and autophagy. The proterome is a complex functional system arranged by the cell to organize its ability in addressing autophagy or apoptosis. Researchers are still wondering how the complex machinery controlling cell cycle and survival works despite the numerous external insults, which also encompass phytotoxic xenobiotics. Usually reactive oxygen species (ROS) and reactive nitrogen species (RNS) are simply considered toxic or potentially damaging waste products, but they actually serve as major signaling molecular species that allow the cell to trigger its survival machinery. ROS are even produced by plant phytochemicals and xenobiotics. From this perspective, we probably have to dismiss some prejudices about the role of antioxidant molecules, reactive oxygen species, cell stress response and survival mechanisms. ROS may help cells to survive. In this sense, the proterome is a macrosystem that should assist external ROS-inducing molecules with their fundamental activity of dampening stress-derived damage. If moderated levels of redox reactive species are essential to the survival machinery of cells, those species should act on the main functional system modulating cell survival. This system might be essentially made by the proteasome, endoplasmic reticulum (ER) and mitochondria, the function of which may be referred to as the PERM system or “proterome” (an acronym of proteasome, ER, and chondriome). The proterome, prior to being demonstrated as an anatomical and dynamic structure, is fundamentally a functional concept. Anyway, the physical concept of the PERM system or proterome might yet lie in the possibility that mitochondria and the endoplasmic reticulum join together and act functionally as a single macrosystem, as ER and mitochondria can form the mitochondria-ER associated membranes or MAMs, modulating calcium signalling and autophagy [1]. These MAMs are important platforms for the bioactivity of many signalling pathways leading to the modulation of mitochondria biology. Therefore, the mitochondria, ER and proteasome may create a single structure (the proterome) that should rule the decision either of autophagosome formation or the apoptosis cascade, besides acting on the mitochondria fission/fusion dynamics [1]. The fundamental role of the proterome or PERM system is to maintain cell survival through its biochemical and functional machinery, which most probably adopts chaotic mechanisms leading to strange attractors. Some evidence reported below would even suggest the physical though transient existence of an intracellular PERM complex, which should activate the mechanisms explained in this review.

**Figure 1:**
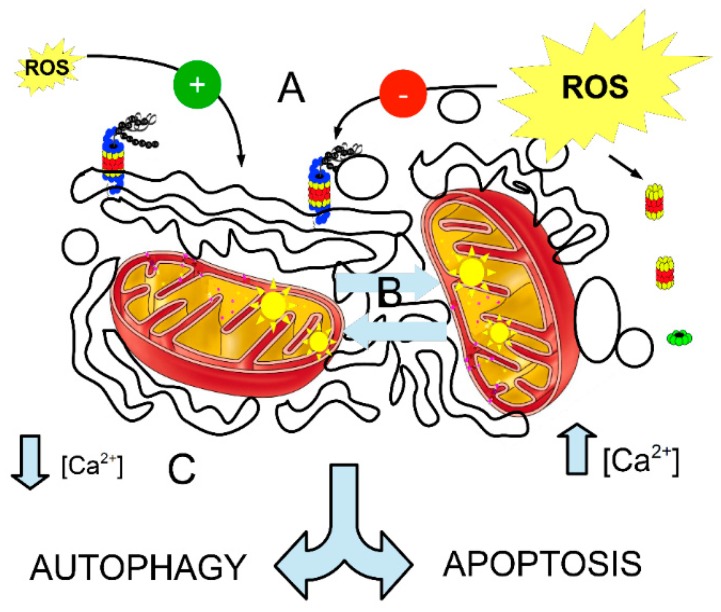
Suggested model for a proterome (PERM (Proteasome, Endoplasmic Reticulum and Mitochondria) system). The endoplasmic reticulum (ER) joins the oscillating mitochondria (the exact topology should be further evaluated) when the three main chaotic oscillators (indicated by letters) synchronize due to (**A**) stressors (ROS) accretion or persistence of the ROS-dependent stress; (**B**) Increase in the percentage of oscillating mitochondria (related to inner membrane Δψ); (**C**) Perturbation in the calcium oscillatory system. In these conditions, it is presumable that a proterome may be created in order to help cells decide between autophagosome production and apoptosis. ER stress should be considered as a “buffer” system, where ROS synchronize with the activity of 26S-proteasomes in order to reduce ER stress. When ER stress overwhelms a threshold value, then 26S-proteasome disassembles. The reduction (scavenging) of ROS is made by the chaotic activity of CYP450 and by a 26S-proteasome (not shown). Most probably, in the usual condition, ER stress is the main trigger of proterome formation. Green positive symbols mean activation or induction, red negative ones are inhibition. The greater the ROS yellow flash, the higher the ROS concentration or persistence. See text for further comments.

The activity of the proterome (PERM system) is summarized in Figure 1.

## 2. Organelles and Signalling Molecules of the Proterome

### 2.1. Role of Flavonoids as Stressing and Signalling Molecules

Both chemical xenobiotics and plant-derived compounds may elicit reactive oxygen species (ROS) in the cell and in doing so they have the fundamental purpose to release ROS into the mainstream of signalling molecules, henceforth activating proterome function. Phytochemicals are plant-derived bioactive molecules, often associated with the term phytoestrogens, phytonutrients or nutraceuticals, which usually share aryl-hydrocarbon structures such as phenolic, polyphenolic or heterocyclic backbones and together with environmental xenobiotics they exert a fundamental action on human health [2,3,4,5]. Some more commonly known phytochemicals, such as flavonoids, have contributed to the scientific literature about the effect of food components on the individual’s machinery devoted to the complex homeostasis of cell survival, energy expenditure and response to stressors, often leading to the overall benefit to the organism. Interestingly, this health-promoting effect may also be exerted by a great deal of chemical xenobiotics at low doses, either as naturally derived compounds or even chemicals coming from industrial pollutants, so expanding the debate in the research community about the actual beneficial mechanism of these substances and the hormetic role of toxic pollutants in human health [6]. Fundamentally, phytochemicals and xenobiotics from human industrial activity should act as potentially toxic and noxious substances, the former because plants produce highly complex biochemical compounds to face other organisms’ insults and predation, the latter because the human body is unable to address the burden of pollutants released from anthropization. What is particularly intriguing is that these substances, particularly if within a defined range of concentration, should act as beneficial rather than toxic molecules. A recent theory suggests that, during the evolutionary course, animals and fungi developed systems able to counteract their toxic or genotoxic action and potentiate cell response to this stress, causing improvement in cell survival, a mechanism that would explain our current ability to respond to industrial pollutants, if at low doses [7,8,9,10,11]. However, the mechanism by which flavonoids act as beneficial substances in animal cells is still far from being fully elucidated.

Most of these phenolic compounds are yet known as molecular inhibitors. In a more general way, phytochemicals inhibit several signaling pathways involved in at least three main mechanisms, i.e., inflammation, cell cycle/apoptosis and redox response/survival, such as PI3K/Akt/mTOR [12,13,14,15,16], MAP kinases (MAPK) [17,18,19], ERK1/2 [20], Wnt/β-catenin [21] and NF-κB [22,23]. It appears quite clear that these substances, which should act as toxic compounds in animals and fungi, as they are produced from plants quite exclusively with a defensive purpose, must inhibit many cellular signalling pathways. Interestingly, most of them exhibit a beneficial action of cell biology [24,25,26,27], apparently despite their toxic nature.

Researchers worldwide are still concerned to elucidate the complex machinery leading to a benefit rather than noxious effect in an organism and to understand its ability to modulate the very fine balance between stressors’ accretion and the stress response aimed at scavenging stressors, when cells are targeted by these plant-derived phenolics or by xenobiotics [28,29,30,31]. Usually, these compounds are considered natural antioxidants and often collected within the same biochemical group that usually includes vitamins [32,33,34]. However, this definition is not perfectly suitable to the huge and highly heterogeneous panoply of substances (>8000) coming from plants, which might even behave as toxic pollutants in their hormetic range [35,36]. Therefore, while their antioxidant ability is a possible hallmark of their biological action, a much more complex role should be associated with these molecules and xenobiotics when dealing with hormesis in cell survival. Plant phytochemicals, and likewise pollutant xenobiotics, are able to target mitochondria as well as the endoplasmic reticulum (ER) and the proteasome detoxifying activity [37,38,39,40,41]. Most probably the proteasome–ER–mitochondria system (PERM) should act as a complex single machinery, where ROS and probably RNS, too, act as signalling modulating molecules. This perspective would suggest that phytochemicals and pollutant xenobiotics, particularly in a defined range of concentrations, activate complex cell machinery devoted to the monitoring of cell survival, made up of the mitochondria–ER (peroxisome) and proteasome, where redox-generated molecules have a major signaling role. If phytochemicals and other external pollutants (xenobiotics) are able to elicit proterome activity, ROS-generating systems, such as peroxisomes, mitochondria and ROS-scavenging systems, within the cells are of major importance to address the ability of cells to respond to stress and switch on survival functions.

### 2.2. Role of Peroxisomes

Both ROS and RNS are usually considered waste toxic species against which proper antioxidant activity and cytochrome-mediated scavenging of ROS-derived stressors should act for cell survival. This is partly true, yet ROS are also fundamental signalling molecules [42,43]. Xenobiotics may elicit ROS, generating exogenous-borne reactive species, which should add to their action with endogenous ROS [44]. In this context a major role is exerted by peroxisomes, which are at the crossroads of the stress response and mitochondrial function [45,46,47,48] and more likely because, at least as observed in plants, peroxisomes participate in regulating ROS and RNS-mediated signalling networks [49]. Furthermore, they even may be a source of ROS as signalling molecules [50]. In mammalian cells, redox molecules from peroxisomes target the mitochondria [51] and therefore a fundamental relationship might exist between redox signalling from the peroxisome and mitochondria. What is well known is that peroxisomes are eukaryotic organelles that contribute to the breaking down of fatty acids (FAs), encompassing both long-chain and branched-chain FAs, for the subsequent β-oxidation in mitochondria and use oxygen to produce ROS and scavenge ROS themselves via the enzymatic anti-oxidant endowment [52]. Lon protease isoforms, LonP1 in mitochondria and LonP2 in peroxisomes, appear to play a major role in this relationship [52]. Lon proteases are a family of proteases that are found in *Eukaryota* but also in *Archaea* and *Eubacteria* and are ATP-dependent serine proteases, belonging to the MEROPS database peptidase family S16, mainly present in the mitochondria matrix [53,54]. Their role in the ROS-signalling pathway and related functions leads to a modulation in the role exerted by mitochondria on cell survival, and thus might be much more intriguing than expected. Mutations of mitochondrial LonP1 may induce the multi-systemic development disorder causing Cerebral, Ocular, Dental, Auricular, and Skeletal anomalies (CODAS syndrome) [55]. The role of these proteases, therefore, might be fundamental as a functional link between peroxisomes and mitochondria. What appears as a fundamental task of mitochondria, in the relationship with peroxisomes, ER and proteasome, is the removal of oxidative modified proteins and damaged proteins, a crucial process to maintain cell survival and homeostasis. Lon proteins appear to have a major role in this mechanism, as Lon levels have been associated with ageing and senescence, where senescent cells often lose their intrinsic ability to induce Lon expression during acute stress [56,57]. It would appear that ROS may act as a signal in this Lon upregulation in response to stress [58], and that ROS-dependent apoptosis is controlled by mitochondrial Lon proteases [59]. Although this evidence was reported to date only in certain cell models, it may serve as a conceptual framework to comprehend how ROS act as signal molecules to elicit protective or pro-survival mechanisms in which mitochondria play a role. The role of peroxisomes in this context is to balance the amount of ROS in order to elicit a cell stress response and the role of Lon proteases might be intriguing, deserving further insights in next future.

### 2.3. Role of the Proteasome

Furthermore, a metabolic interplay exists between peroxisomes and other intracellular organelles, whereby peroxisomes are involved in the ROS/RNS metabolism [60]. Reactive nitrogen species (RNS) participate with ROS to create proteomic homeostasis, and an imbalance in the redox state may disrupt the proteostasis network [61]. If the activity of ROS as signalling molecules may target the PERM system, then activities of ROS should be shown in the macromolecules participating in the same system. Oxidative stress regulates proteasome activity [62]. In the model proposed by Aiken et al., the mild stress condition should activate 26S proteasomes while with persistent ROS-dependent insult, the proteasome should disassemble into 20S core particles (CPs) and 19S regulatory particles (RPs) [62,63]. When this dissociation occurs, free 20S proteasomes, which are henceforth activated, will degrade oxidized proteins via a pathway working independently from the involvement of ubiquitin and ATP [62]. Yet, the relationship between ROS and ubiquitin–proteasome systems (UBS) is not a true novelty [64,65,66,67]. ROS can modify some reactive Cys residues (i.e., Cys273, Cys288 and Cys151) in the Kelch-like ECH-associated protein-1, known as Keap1, a substrate adaptor protein for a cullin3-dependent E3–ubiquitin ligase complex, so leading to the inhibition of the ubiquitination and degradation of Nrf2, IKKβ and Bcl2/Bcl-xL [64]. The role of 26S proteasome is intriguing in this context. This 25 MDa protein macrocomplex, made up of at least 31 subunits, finely regulates protein degradation [68]. The 26S proteasome disassembles upon mitochondrial stress [69]; conversely, the mitochondria participate in regulating the activity of the proteasome [70]. In this sense, the proteasome is finely linked with activity in response to mild stress, which should activate a higher degradation of unfolded or damaged proteins and allow cells to maintain their functional integrity.

### 2.4. Role of Mitochondria

The role of mitochondria in this context is of the utmost importance. Many aspects will be discussed below. Mitochondria turnover and homeostasis, particularly the fission and fusion mechanism, are strictly associated with the proteasome activity. The homeodynamic balance between fission and fusion is a fundamental hallmark of cell survival and protein homeostasis. The interaction between the mitochondria outer membrane molecule mitofusin-1 (Mfn1) and the ubiquitin E3 ligase Mahogunin Ring Finger-1 (MGRN1) is, for example, critical for cell function, as defects in this mechanism, such as those caused by the absence of MGRN1, lead to neurodegenerative pathologies [71]. This would mean that the participation of the ubiquitin–proteasome system in the mitochondria biogenesis and homeostasis represents a crucial function in the apoptotic vs. autophagy balance [72,73,74]. Therefore, reactive species coming from oxidative stress may be particularly involved in the peroxisome–ER–mitochondria relationship [75]. Mitochondria might be the functional engine of the proterome. Their ability to create an oscillatory system, described below, gives the opportunity to the chaotic system made by those organelles involved with ROS balancing, i.e., peroxisomes, endoplasmic reticulum (ER) and the proteasome, to give cells their chance to choose autophagy (mitophagy) or apoptosis, through the activity of the proterome.

### 2.5. Role of the Endoplasmic Reticulum (ER)

The endoplasmic reticulum (ER) plays a major role in controlling and regulating the dynamics and amount of ROS. Furthermore, ER plays a major role in the introduction of calcium in the proterome activity. The fundamental source of the intracellular calcium signalling is the endoplasmic reticulum (ER), which therefore participates actively in this dynamics, even involving mitochondria [76]. The role of the ER is of major importance in controlling the switch to autophagy rather than apoptosis, behaving as a master tuner of this bifurcation, principally thanks to calcium. The role of ER in the apoptosis/autophagy balance, i.e., in that critical decision-making that cells exert in order to die or survive, has been actively investigated in certain critical models, such as the hypoxia/reperfusion injury [77]. In this model, the proteasome is also involved. The inhibition of the 26S proteasome strengthens autophagy but increases ER stress while reducing both apoptosis signals and the inflammatory response. A very similar effect is obtained if simply promoting autophagosome formation [77]. This would suggest the existence of an interesting link between ER stress and autophagy, i.e., an inhibition of the autophagic signals, while existing ER stress (probably through a PERK or IRE-1-dependent mechanism) should lead to apoptosis with theaid of calcium signaling [78]. Both ROS and cell calcium signals regulate the activity of the proterome, in order to shift cell homeostasis to the survival response (autophagy) or the death response (apoptosis), this latter to prevent further damage to an organ or the whole organism.

The activity of ROS on ER may lead to ER stress, mitochondria dysfunction and apoptosis cascade, for example by triggering the activity of ER stress markers such as 78-kDa glucose-regulated protein (GRP78), caspase 12 and C/EBP-homologous protein (CHOP), resulting in a mitochondria-mediated apoptotic signaling. This cellular “choice” is most probably the effect of an ROS-mediated imbalance in the proteostasis, i.e., the homeostasis of the protein degradation/synthesis, as occurs in cancer cells, where notoriously antioxidant substances, such as the stilbene resveratrol, if associated with a chemical xenobiotic, lead to the apoptotic event [79]. The question is whether this ER stress possesses some functional “threshold” or “switch tuners” for which cells decide to suicide through a damaged mitochondrial endowment or not. A possible answer should come from those tumour cell lines expressing a methotrexate resistance phenotype. In these in vitro systems, cells rescued their survival property through the PERK/ATF4 axis via the ER stress, which shifted the apoptosis/autophagy balance towards the latter. The “switch” was performed by the JNK/p62 signalling pathway [80]. The endoplasmic reticulum has two major resident proteins, namely PERK and IRE-1, the sustained activation of which leads to two different and quite opposite functions—apoptosis or cell proliferation, respectively—probably through a calcium overload and the Ca^2+^ transient signalling between the ER and mitochondria [81,82,83].

## 3. Proterome Function

### 3.1. The Role and Activity of the Proterome in the Autophagy/Apoptosis Balance

How does the proterome work? ROS are produced by various sources, either physical or chemical in nature. In summary, the role of the proterome is to dynamically regulate the chaotic mechanism leading to either autophagy or apoptosis. It uses ROS as signalling molecules and controls their ability to induce mild stress by acting on the chaotic system generated by mitochondria, ER and the proteasome and fed by the oscillatory system generated by the mitochondria and calcium. Autophagy dampens ER stress through the clearance of ubiquinated proteins and switching off the pro-apoptotic signal generated by too much ROS or an impaired calcium efflux. Factors causing early ER stress increase the expression of p62, then cause its downregulation through the increase of 78 kDa glucose-regulated protein (GRP78), microtubule-associated protein1 light chain3 (LC3), inositol requiring enzyme1 (IRE1), IRE1α, beclin and also p-JNK, leading to autophagy via the regulatory pathway IRE-1/JNK/beclin [84]. Actually, autophagy is an alternative way of programmed cell-death and ER stress is the real master tuner, able to switch on autophagy or apoptosis depending on the mitochondrial stability. Some signalling pathways serve this purpose; for example, ER stress leads to autophagosome formation, involving LC3 conversion via either PERK signalling (for example, the PERK/eIf2a pathway) or the JNK pathway (i.e., the IRE-1/JNK pathway) [85]. In this scenario, the role of tuners is most probably exerted by two major pathways, IRE-1 and PERK. Inositol-requiring enzyme 1 (IRE-1) modulates ER stress in its “protein” face, namely the ER transmembrane sensor that activates the unfolded protein response (UPR) [86]. UPR generates ROS and, if not properly and fully addressed, leads to apoptosis.

The role of ROS in this context is therefore fundamental.

**Figure 2:**
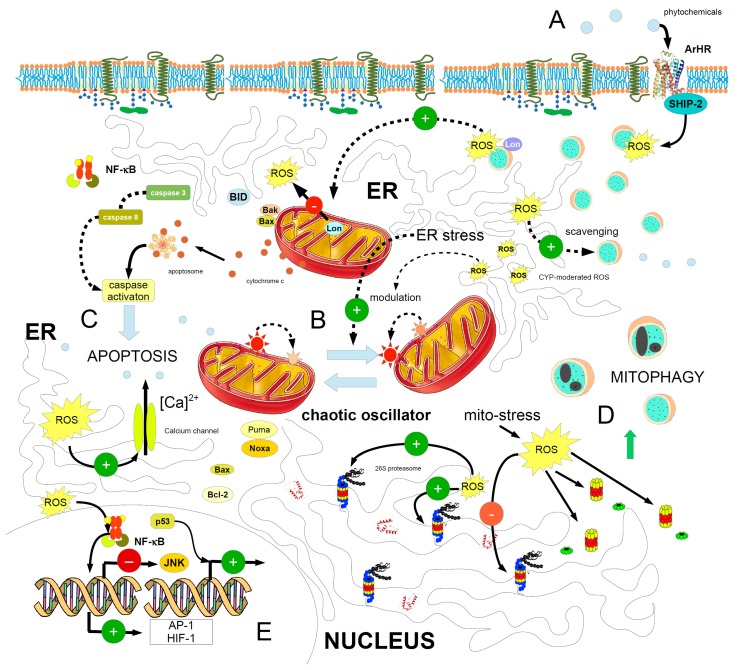
The major pathways and mechanisms of the cell stress response to phytochemicals and existence of the proterome. (**A**) The phytochemical targets an aryl hydrocarbon receptor (ArHR) and generates ROS through a signalling involving membrane, SHIP-2; lysosomes are the source of ROS, as signal molecules. Lysosome Lon proteases activate mitochondrial Lon homologues, which dampen ROS production from the mitochondria; (**B**) Mitochondria form a chaotic oscillator through their inner membrane potential, which is initially modulated and maintained (through a synchronization) by ROS of ER stress, which also activates the scavenging of stressors by lysosomes. This is a major hub in the cell homeodynamics, because the choice between mitophagy and apoptosis depends on the many factors (discussed in the text) unbalancing this ER stress/mitochondria relationship; (**C**) Shifts in the oscillatory mechanism of the intracellular calcium, in a persistent high presence of ROS, may lead to apoptosis; (**D**) Stress accretion and a high amount of ROS leads to the proteasome breaking down, with release of the 20S and the regulatory units and activation of the autophagy; (**E**) Genetic control of ROS. Green positive symbols mean activation or induction; red negative ones mean inhibition. The greater the ROS yellow flash, the higher the ROS concentration or persistence. Dashed arrows: activity to be assessed (hypothetical or not yet fully elucidated). Sign plus = activation; sign minus = inhibition. See text for details.

This quite complex scenario would suggest that ROS are most probably used as “calibrators” or as the “checks and balances” adopted in a steelyard to allow cells to maintain survival functions and decide to turn on autophagy of damaged organelles (mitophagy, for example) or apoptosis, through the ER stress and mitochondria impairment, which drives and modulates the activity of proteasome. The actual messenger in this system should be ruled by the oscillatory peaks of the intracellular calcium [87,88,89,90]. Figure 2 summarizes the main biomolecular mechanisms involved in the mechanism. The fundamental engine of this complex machinery might be some mechanism underlying the synchronization of mobile chaotic oscillator networks, or at least their interaction [91]. It is tempting to speculate that the proterome should self-organize when ER stress reaches a critical cutoff point, and calcium signalling would probably elicit, and then enhance, this mechanism. Anyway, besides calcium, ROS also participate within the cell in the building up of an oscillatory network, involving mitochondria [92,93]. This oscillatory behaviour, linking ROS and mitochondria, is also associated with the circadian rhythms [94], therefore making this cellular mechanism fundamental to cell survival and growth. ROS have recently been mathematically modelled as fundamental elements of a two-dimensional network, interacting with the oscillatory mitochondria network. This would mean that, when stress occurs, causing an abrupt collapse of the PERM homeostasis or oscillations of the mitochondrial biodynamics, their energy state is synchronized through the mitochondrial oscillatory network by local interactions dependent on ROS [95,96]. These mechanisms suggest the presence of a self-organized macro-structure, such as the proterome. Table 1 summarizes the main chaotic mechanisms underlying the function of the proterome in the context of cell stress response and its relationship with phytochemicals.

### 3.2. Activity of the Proterome: ROS as Signalling Molecules and the Role of Xenobiotics

It is widely accepted that the effect of plant-derived chemicals, also called phytochemicals, and low-dosed chemical xenobiotics is to promote cell response to stress, thereby eliciting a positive and beneficial outcome on cell survival. Furthermore, both phytochemicals and xenobiotics are able to produce ROS. In this PERM hypothesis these compounds might act as “perturbing agents” of the chaotic oscillatory systems driving cell homeodynamics to reach its functional attractor, which should lead either to survival pathways, autophagy (mitophagy) or apoptosis (see also above). We have suggested that this machinery adopts both calcium and ROS as important signalling mediators. Phytochemicals may enter the cell principally through the aryl-hydrocarbon receptor (ArHR) [106,107]. The relationship between ArHR and ER stress has recently been reported in a mast cell model, where the activation of ArHRs with ligands led to ROS and calcium signalling. Calcium peaks in the oscillatory system caused by the ArHR activation are regulated by the SHIP-2 phosphatase, which therefore is critical for the activation of the ER stress and intracellular calcium, in response to the ArHR-mediated signalling [108]. In this model, a ligand of ArHR activates SHIP-2, which in turn promotes, via ROS-mediated signalling, an ER stress response that involves the PERK signalling pathway and also the ATF4 activation and eIF2α phosphorylation [108]. This is most probably a “perturbing signal” caused by the ArHR ligand to the PERM chaotic dynamics. Actually, this perturbation seems to be “restored” by the activity of *N*-aceyl-l-cysteine (NAC), a well-known antioxidant [108].

As ligands of ArHR are also antioxidant molecules, this mechanism merits further discussion. The mast cell model shows that ligands of ArHR initially cause a calcium signal, then a ROS signal and an ER stress response. In the PERM hypothesis we suggested here, the ArHR ligand “perturbs” the homeodynamic machinery made by the activity of the proteasome, ER and mitochondria, where ER can be considered the dynamic pivot of the system. Probably, phytochemicals are good inducers of the proterome formation. The proterome thus has to be considered as a single functional macrosystem in the cell, able to drive cells to pro-survival decision-making. Actually, the antioxidant effect in normal cells or the pro-apoptotic effect in tumour cells seem to confirm this suggestion, i.e., cancer cells most probably lose their ability to respond to stress and ER stress leads to the apoptotic event [109]. It is intriguing that, while relatively low doses of xenobiotics, radiation and phytochemicals activate the survival machinery in normal, healthy stress-responding cells, those doses are able to trigger apoptosis in cancerous ones. The concept of “hormesis” has been suggested to explain how neurotoxicants, pollutants or genotoxic substances, able to trigger a redox signaling and ROS production, exert a beneficial action on cells in certain doses or a pre-conditioning state [110,111,112,113]. As these substances are intrinsically noxious, as plants produce them as a defensive mechanism against predators and pollutants are rarely common in the body’s original composition, then their beneficial or detrimental activity depends only on the system homeodynamics or, otherwise, on the system’s chaotic behaviour with its bifurcation.

### 3.3. Mitochondria in the Proterome Activity

It is arguable that the critical step in the PERM system may be represented by the ER stress, to which the proteasome activity should also be related. A possible factor able to increase the activity of proteasome under ER stress condition is the inactive rhomboid protein 1 (iRhom1), which is known also as rhomboid 5 homologous 1 or rhomboid family member 1 (RHBDF1) [114]. Stressors able to induce ER stress are also able to enhance iRhom1 expression and activity, thus leading to its interaction with the 20S proteasome associated ER-resident chaperones PAC1 and PAC2 and increasing proteasome activity [114]. Rhomboid proteases are probably the most intriguing recent targets of our investigation on PERM function. Their activity might be fundamental to elucidating the activity of PERM or the proterome. Besides the above mentioned iRhom1, mitochondria also have rhomboid proteases, called presenilin-associated rhomboid-like protease or PARL proteases, which should accomplish complex functions associated with the maintenance of the mitochondria steady state and homeostasis, related to the stress response [115,116]. The role of PARL proteases is fundamental for the regulation of mitochondria function and stress response, as PARL is a master tuner of the mitochondria’s ability to respond to stress [116]. Therefore, the newest suggestion for PERM activity includes the role of rhomboid proteases. These molecules should serve as fundamental switching tuners to apoptosis or autophagy in the PERM function. PARL activity is also involved in the mitophagy process [98]. When mitochondria have a high Δψ_m_, as occurs in polarized mitochondria with preserved mitochondrial potential, usually PARL, which is located in the inner mitochondrial membrane, cleaves the serine/threonine kinase PINK1, which is imported through the TOM/TIM complexes, and probably also cleaves PGAM5 and OPA1 [115]. Notably, PGAM5 is an AIF-associated factor and triggers caspase activation and cell death [99], and OPA1 controls cristae remodelling during apoptosis; however, what is very interesting is that OPA1 belongs to a wide group of proteins (mitofusins, proteins of the Kif family, Bax, Fis1 and Drp1) that link the chaotic oscillatory mechanism of the mitochondria fission/fusion with processes altering the mitochondrial membrane potential, leading to apoptosis if dysregulated and autophagy if finely regulated [100]. When mitochondria have a low Δψ_m_, i.e., in depolarized mitochondria or particularly when mitochondrial potential collapses, PARL is unable to cleave PINK1, as it is no longer transported into the membrane by the TOM/TIM system, leading to PARKIN recruitment into the cytosol and the activation of mitophagy, in order to eliminate damaged mitochondria [115,117]. The system suggests that membrane potential cannot be the only trigger able to lead cells to the apoptotic cascade, as mitochondria are mainly induced to be autophagocyted, prior to leading to cell death by an apoptotic mechanism.

Substances inducing ER stress are fundamental in this dynamics.

### 3.4. Calcium Oscillation and Proterome Chaotic System

The model presented here suggests that ROS, particularly if eliciting a mild stress, are controlled by the ER activity, in conjunction with peroxisomes and mitochondria, and modulating the activity of proteasome, in order to maintain cell survival, often leading to an autophagic mechanism to safeguard cell integrity. Therefore, in this model, the self-assembly of the proterome depends on the ER stress/mitochondria relationship, which is often regulated by ROS.

The flavonoid interacting with the ArHR should induce an initially attenuated ER stress, which can cause a moderate loss of the mitochondrial Δψ. The overexpression of Bcl-2, together with a deficiency in caspase 9 and caspase 2, reduces this ER stress-mediated loss [101]. ROS activate the 26S-proteasome and this should induce an increase in the level of Bcl-2, as previous data showed that the inhibition of 26S proteasome induces a decrease in Bcl-2 [97]. The role of Bcl-2 may be intriguing in this context: cancer cells are often dysregulated in the apoptotic response but the persistently low Bcl-2/Bax ratio might even promote the pro-apoptotic effect of phytochemicals. Conversely, the inhibition of proteasome also leads to apoptosis, a mechanism that may explain the role of p53 and the consequent upregulation of PUMA and Bim [118].

Yet, the scenario is most probably much more complicated than that.

The moderated stress response also generated the concept of “mitohormesis” [119,120]. Mitochondrial hormesis is represented as a non-linear response to ROS, which are considered well-known signalling inputs, even in plants [121,122].

In the PERM mechanism, the production of ROS and the intracellular calcium overload should lead to the depolarization of the mitochondrial inner membrane potential (low Δψ_m_) and subsequent cell damage. How can this mechanism, involving as it does only a few mitochondria (mitohormesis), drive all the cells to a fatal or survival decision? In cardiac myocytes, where the chaotic oscillations are more evident, it was reported that synchronized and self-maintaining oscillations occur in the Δψ_m_ of the inner membrane and involve ROS and NADH in at least 70% of the chondriome, i.e., almost the total endowment of active mitochondria in the cell [123]. These oscillations, which could be synchronized, started after about 40 s of lag time, i.e., after a specific threshold level of mitochondria-produced ROS was exceeded [123]. Initially, this mechanism does not involve calcium overload. Therefore, this assesses the hypothesis that a threshold exists, at least in terms of ROS and that calcium enters the system in a second time. Actually, at least in the cardiomyocyte model, only 0.3% of mitochondria initially depolarize, creating a local perturbation in the functionality of mitochondria. This local perturbation, triggered and maintained by ROS, generates an oscillatory mechanism, depolarization/hyperpolarization, in a synchronized manner, after a lag time, probably depending on the exceeding of the ROS threshold [123]. In this condition, the simple existence of an independent mitochondrial functional network should lead to rapid cell apoptosis, if this network is not modulated and buffered by the relationship with proteasome and ER stress. This might partly explain why toxicants such as heavy metals, xenobiotics or phytochemicals act in pro-apoptotic, pro-oxidant compounds when they are at a high dosage. During this oscillatory mechanism, the proteasome contributes to the regulation of the process through the activity of parkin, a ubiquitin E3 ligase. In dysfunctional mitochondria, when oscillations are synchronized to lead to a greater number of depolarized mitochondria, parkin activates the ubiquitin–proteasome system (UPS), which induces the proteolysis of the outer membrane proteins of mitochondria and leads to mitophagy [124]. The same parkin is able to promote the degradation of ARTS, known also as Sepr4_i2, a mitochondrial protein that may initiate the apoptotic cascade through the activation of caspases upstream from the cytochrome c release [125].

Calcium is in this context most probably used to irreversibly turn cells to an apoptotic decision. We are yet unable to verify if calcium oscillations may inter-relate with other oscillatory systems, such as mitochondria or ROS/proteasome, to modulate cell decision making. This should occur particularly when stressors overwhelm the chaotic mechanism of intracellular balance ruled by the proterome. The system as a whole should therefore work as a dynamical chaotic oscillator. Synchronization is a classical non-linear phenomenon, occurring widely in biology and chaotic oscillators can be studied by the concept of phase applied to the case of continuous time-chaotic systems. Moreover, any type of synchronization can be considered, in a general way, as the appearance of some additional order inside the dynamics. For chaotic systems, this would mean that the dynamics in the phase space is restricted to a symmetrical sub-manifold. Therefore, only from the point of view of the topological properties of chaos, the synchronization transition usually means that simplification leads to the structure of the strange attractor. Chaotic systems having a strange attractor structure promote new synchronization with other systems [126,127,128].

Despite the fact that the proterome or PERM system is still speculative, many recent findings suggest the existence of a macrostructure linking the mitochondria, ER and proteasome, adapted to respond to stress in a chaotic oscillatory synchronization and leading cells to their major decision, usually translated into an autophagic or apoptotic event.

### 3.5. Chaotic Activity Elicited by ROS in the Proterome

In this scenario, the early pro-oxidant activity exerted by xenobiotics and phytochemicals may give important insights into the issue, probably contributing to elucidate the complex mechanism underlying how cells control redox signaling. The commonest opinion is that flavonoids act as pro-oxidant molecules at a very high dosage and that this activity could be related to their chemical flavone and/or polyphenolic backbone and chemical substituents [127]. Our opinion is that, in the context of the U-shaped curve of the hormetic dose response, these phytonutrients act as mild pro-oxidant molecules at relatively low dose ranges (picomolar–micromolar), as antioxidants at sub-micromolar/micromolar ranges (the U-bottom) and as pro-oxidants at high dosages (micromolar-millimolar). Actually, the pro-oxidant activity of flavonoids has recently been reviewed [128]. Xenobiotics and phytochemicals have, therefore, a pro-oxidant activity [102,129,130]. In the so-called “Murburn hypothesis”, Manoj et al. reported that small amounts of diffusible reactive oxygen species or DROS are fundamental to making the CYPs machinery involved in the redox metabolism of liver microsomes work [129], hence the generation of small amounts of ROS within the cell is required for the housekeeping metabolism [129]. Manoj and co-workers stated that the mechanism involving these species is chaotic [129]. In this chaotic system, DROS may work aside from the catalytic centre of detoxifying enzymes, i.e., there is no obligation for a substrate or an inhibitor to react with the heme centre and influence the overall mechanism of catalysis, and in this sense ROS are not only substrates but finely acting regulators of the cytochrome-redox activity [130]. A chaotic behaviour was reported in past years on peroxidase [102,131] and some researchers suggested that an increase in the concentration of reacting species should lead to imperfectly mixed systems with both stochastic and chaotic behaviour [130]. If the system underlying the redox machinery is made of a particular topology of the network built up by different switches with fluctuating response times, then it may lead to a defined cell activity. This event probably depends on the “noisy” level of ROS signalling, i.e., when the elements making the network functional tend to synchronize by suppressing fluctuations, they lead to reliable dynamical attractors [132]. In other words, the mild, finely regulated working of routinely produced ROS may lead to a chaotic system that is synchronized by an external factor to reach a reliable dynamical attractor, i.e., an irreversible cell response. Our hypothesis is therefore that ROS are finely tuned to maintain a chaotic oscillatory system, synchronized with mitochondria and calcium, in order to allow the cell to stay in a homeodynamic state, which should let it respond promptly and properly to any stressful stimulus. Proterome makes this possible. On the other hand, an unreliable dynamical attractor may come when networks desynchronize, as may occur when the chaotic system changes to a more stochastic system, i.e., when the concentration of the reactive species is too high or persistent due to defects in the scavenging systems [102,132]. Very recently, the hypothesis of so-called “mild oxidative stress” should explain the role of ROS in sperm telomere length maintenance, showing a delicate balance in the dynamics of telomere structure by ROS levels [133,134,135]. In this context, ROS, induced by physical or chemical stress, are formidable signalling molecules of the homeodynamic activity of the proterome.

The “PERM hypothesis” we are formulating here, while suggesting the existence of a new macrosystem called the proterome, starts from the fundamental consideration that ROS are major signalling molecules in the regulation of the survival process of the cell [136]. Therefore, paradoxically, this seems to be the main functional role of ROS, though they are also waste by-products of protein turnover and the mitochondria redox mechanism. Cells use ROS as subtle signals of the many inputs coming either from outside or inside the cell, even electromagnetic fields [103,137]. Their relationship with mitochondria is particularly intriguing.

An emerging functional relationship between ROS or oxidative stress, mitochondria and autophagy has recently been reviewed [138]. This relationship should also shed a light on mitochondria-triggered apoptotic mechanisms. The early generation of ROS is a critical step for mitochondrial damage, which may activate the intrinsic apoptotic pathway, but early ROS can be regulated by autophagy [139]. Both chemical and physical stressors, such as electromagnetic fields and ionizing radiation, generate ROS but also elicit a panoply of signalling molecules that regulate autophagy, i.e., PARP1, sirtuins, FOXO-3a, ATM and mTOR, and increase ceramide and intracellular calcium [104]. Initially, ROS should interact with the PERM system through the peroxisome’s calcium signalling. It is well known, although still under investigation in plants, that peroxisomes “sense” the chaotic level of ROS/RNS and other nitrogen-related species, such as NO, and act as decision-making platforms to adjust cell function towards the most correct and proper response to stressors [49]. Peroxisomes themselves produce ROS as signal molecules [47,50]. The initial triggering of the calcium signal most probably switches on the starting engine of the complex interplay between calcium and ROS, two master tuning systems within the cell, both chaotic and oscillatory, which modulate many important signalling pathways [105].

## 4. Conclusions

PERM is a functional terminology to indicate a complex dynamical system made up of the proteasome, endoplasmic reticulum (ER) and mitochondria, which has been called the proterome. Most probably this system acts as a single master tuner of cellular decision-making about life or death, in order to give a chance to the remaining living cells in an organism. The ability to adapt its dynamics to the huge number of stressors, insults and stimuli may lie in its chaotic behaviour, mainly formed by synchronized oscillatory mechanisms, which involves several important components of the proterome, such as calcium, ROS and mitochondria polarization of the inner membrane. The synchronization of at least two chaotic oscillatory systems should lead to a bifurcation point that results in a final functional action, usually divided between the decision to eat damaged organelles (mitophagy) and hence survive, or to die (apoptosis). Many interesting and newly discovered proteins participate in this modulation. Xenobiotics and phytochemicals can perturb this modulation and lead to cell injury when the system is dysregulated (cancer or damaged cells) or when the stimulus is exacerbated or persistent, while, under a threshold level, they act indirectly, potentiating the ability of the PERM system to counteract damaging signals.

This overview of mitochondria as a component of a more complex system including the proteasome and ER may improve our understanding of the role and activity of flavonoids and xenobiotics in human health, though further experimental investigations are needed to assess and improve the elucidation of these models.

## Figures and Tables

**Table 1:** Chaotic behaviour of some signalling pathways and macromolecular systems in the activity of proterome.

System	Description	Working Structure	References
CYPs-ROS	Murburn hypothesis	Small amounts of ROS are able to switch on the chaotic network of cytochrome P450 groups	[61,62]
ROS-mitochondria	Chaotic synchronization of oscillation networks	The macroscopic property of the mitochondrial network is reproduced in a reaction-diffusion model of ROS-induced ROS-release	[97]
ROS-calcium	Chaotic interplay	Sub toxic levels of ROS interplay with calcium signaling network	[92]
	Calcium oscillations	On the basis of the permeability of the ER channels and on the kinetic properties of calcium binding to the cytosolic proteins, different patterns of complex calcium oscillations occur	[47]
Proterome	Chaotic synchronization	Synchronization of mobile chaotic oscillators in the bi-dimensional landscape	[98]
	ROS signalling	Participation in the synchronization process	[99,100,101]
	Mitochondria	Dynamics in the network	[102,103,104]
	Proteasome and chaperones	Chaotic-type oscillatory system depending of ATP levels	[105]
